# Genetic Variations and Frequencies of the Two Functional Single Nucleotide Polymorphisms of *SLCO1B1* in the Thai Population

**DOI:** 10.3389/fphar.2020.00728

**Published:** 2020-06-05

**Authors:** Chalitpon Na nakorn, Jariya Waisayarat, Charungthai Dejthevaporn, Pornpen Srisawasdi, Sansanee Wongwaisayawan, Chonlaphat Sukasem

**Affiliations:** ^1^ Program in Translational Medicine, Faculty of Medicine Ramathibodi Hospital, Mahidol University, Bangkok, Thailand; ^2^ Laboratory for Pharmacogenomics, Division of Pharmacogenomics and Personalized Medicine, Somdech Phra Debaratana Medical Center (SDMC), Faculty of Medicine Ramathibodi Hospital, Mahidol University, Bangkok, Thailand; ^3^ Department of Clinical Pharmacy, Faculty of Pharmaceutical Sciences, Prince of Songkla University, Songkhla, Thailand; ^4^ Department of Pathology, Faculty of Medicine Ramathibodi Hospital, Mahidol University, Bangkok, Thailand; ^5^ Division of Neurology, Department of Internal Medicine, Faculty of Medicine Ramathibodi Hospital, Mahidol University, Bangkok, Thailand

**Keywords:** pharmacogenomics, single nucleotide polymorphisms, *SLCO1B1*, organic anion transporting polypeptides, OATP1B1, frequencies, haplotypes, Thai population

## Abstract

**Aim:**

To investigate the variations and the frequencies of the *SLCO1B1* gene in the Thai population.

**Methods:**

Collected samples were categorized into five regions of Thailand. DNA samples were genotyped for two variants, c.388A>G and c.521T>C of the *SLCO1B1,* using TaqMan^®^ real-time PCR.

**Results:**

The minor allele frequencies (MAFs) of two single nucleotide polymorphisms (SNPs) were not significantly different among the five regions. The most frequent haplotype was SLCO1B1*1b (frequency: 0.654), followed by *1a (frequency: 0.217), *15 (frequency: 0.128), and *5 (frequency: 0.001). We observed a similar frequency of OATP1B1 transporter phenotypes compared to other populations. 75.85% of the Thai subjects showed normal OATP1B1 activity, 22.5% showed intermediate OATP1B1 activity, and 1.58% showed low OATP1B1 activity.

**Conclusion:**

This study reported the frequencies of the *SLCO1B1* variants and the subsequent OATP1B1 activity in a large cohort of Thais that can provide important information for the guidance of personalized drug therapy.

## Introduction

The transmembrane protein transporters can be divided into two groups, the solute-linked carrier (SLC) superfamily or known as influx transporters, which uptake the substrate through the cells, and the ATP-binding cassette superfamily (ABC) or efflux transporters which pump the substrates out of the cells (Gong and Kim, 2013). Several groups of the influx transporters uptake a variety of drugs and organic compounds from the blood into the cell, especially, the organic anion transporting polypeptides (OATPs) which are expressed in many organs such as the intestine, liver, and kidneys. OATPs have shown an important role in clinical implications for the pharmacokinetics of many drugs, including drug absorption, distribution, and elimination (Gong and Kim, 2013; Maeda, 2015; Alam et al., 2018). Evidence of changes in OATP transport function has been found to affect the efficacy and safety of many drugs and leading to instability in drug disposition and response (Gong and Kim, 2013; Shitara et al., 2013).

The genetic variations in the *SLCO1B1* gene, located on chromosome 12p12.1 and encoding the OATP1B1 influx hepatic transporter, have been widely studied in diverse populations (Ghatak et al., 2010; Mastaglia, 2010; Hu et al., 2012; Mastaglia and Needham, 2012; Sirtori et al., 2012; Shitara et al., 2013; Ramsey et al., 2014; Muntean et al., 2017; Alam et al., 2018). OATP1B1 is a transmembrane protein expressed on the basal side of human liver cells, which not only regulates numerous endogenous compounds, including bilirubin, estradiol, and leukotriene C4, but it also removes many drugs, such as HMG-CoA reductase inhibitors (statins), angiotensin II receptor antagonists (ARBs), angiotensin-converting enzyme inhibitors (ACEIs), rifampicin, some antidiabetic drugs, protease inhibitors (PIs), and some chemotherapies from the blood into the hepatocytes, then metabolizes and removes out of the body (Hu et al., 2012; Gong and Kim, 2013; Maeda, 2015; Alam et al., 2018). The polymorphisms of the *SLCO1B1* gene not only affect the expression, localization, and function of the OATP1B1, but also the drug disposition (Gong and Kim, 2013; Shitara et al., 2013).

Various studies have revealed the single nucleotide polymorphisms (SNPs) of the *SLCO1B1* gene reducing the functional transport activity of OATP1B1 and causing some adverse drug reactions (ADRs) (Mastaglia, 2010; Mastaglia and Needham, 2012; Gong and Kim, 2013; Shitara et al., 2013; Maeda, 2015; Alam et al., 2018). *SLCO1B1* variants, including SLCO1B1*1a, SLCO1B1*1b (c.388A>G, N130D), SLCO1B1*5 (c.521T>C, V174A), and SLCO1B1*15 (c.388A>G and c.521T>C) have been reported in affecting the transport activity and drug disposition (Kim et al., 2007; Ho et al., 2008; Gong and Kim, 2013; Ramsey et al., 2014; Namgoong et al., 2015). Statin-taking patients with *SLCO1B1* polymorphisms showed the area under the plasma-time curve (AUC) up to 130% higher than the patients without the *SLCO1B1* polymorphisms (Ghatak et al., 2010; Sirtori et al., 2012). Also, a reduced transporter activity caused more susceptible to statin-induced myotoxicity in the group of patients carrying *SLCO1B1* polymorphisms than those without polymorphisms (Sakamoto and Kimura, 2013; Hamann et al., 2013; Ramsey et al., 2014). SLCO1B1*5 has been strongly associated with myopathy among the simvastatin users with an odds ratio (OR) ranging from 4.5 in heterozygotes to 16.9 in homozygotes (Link et al., 2008; Ghatak et al., 2010; Mammen and Amato, 2010; Sirtori et al., 2012; Hu et al., 2012; Rallidis et al., 2012; Sathasivam, 2012; Gong and Kim, 2013; Bhardwaj et al., 2013; Dandona, 2014; Albayda and Christopher-Stine, 2014; Maeda, 2015). Also, SLCO1B1*15 showed over 70% reduction in the transport activity compared with wild type and showed the association with myopathy in patients taking pravastatin and atorvastatin (Ghatak et al., 2010; Sirtori et al., 2012; Gong and Kim, 2013; Shitara et al., 2013; Maeda, 2015).

Currently, there is a lack of studies reporting the frequency of the SNPs of *SLCO1B1* in the Thai population. The translational decision in the clinical practices has always used the data from the reports in other populations, the Han Chinese population, for instance. The objective of this study was to investigate the regional frequencies of the two functional SNPs of *SLCO1B1* in the Thai population. The findings of this study will serve as the Thai pharmacogenetic data source for decision in drug therapy in a specific group of patients, especially, in patients who will be treated with statins or other medications that are affected by these genetic variants.

## Materials and Methods

### Samples

In the present study, we enrolled 1,205 samples from the previous cohort of Wongkittichote et al. (2013), which were collected from August 2008 to March 2009 by the Health System Research Institute. The selected samples were then categorized into five regions of Thailand, including Northern, Northeastern, Central, Southern, and Bangkok.

### Genotyping Analysis

Genotyping of *SLCO1B1* polymorphisms was performed using allele-specific TaqMan^®^ MGB probe 5' nuclease assay with real-time polymerase chain reaction (PCR) ViiA7™ system (Applied Biosystems, Life Technologies). The allele-specific TaqMan^®^ MGB probe 5' nuclease chain reaction assay was performed with primers of *SLCO1B1* c.388A>G (rs2306283; on reference sequence NM_006446.4, assay ID: C:_1901697_20) and c.521T>C (rs4149056; on reference sequence NM_006446.4, assay ID: C:30633906_10). Each 6 μl of PCR mixture contained 2 μl of genomic DNA in a concentration of 5 ng/μl, 2.5 μl of TaqMan^®^ Genotyping Mastermix, 0.25 μl of allele-specific TaqMan^®^ MGB probe and sequence-specific primer kit, and 1.25 μl of DNase-free water. The thermal cycler program started with 10 min at 95°C, followed by 50 cycles of 15 s at 92°C and 90 s at 60°C. The allelic discrimination plot was analyzed by ViiA7™ software (Applied Biosystems, Life Technologies). Allele 1 was labeled with VIC^®^ dye fluorescence, and allele 2 was labeled with FAM™ dye fluorescence.

### OATP1B1 Phenotypes Based on *SLCO1B1* Genotypes

The Clinical Pharmacogenetics Implementation Consortium (CPIC) Guideline for *SLCO1B1* and simvastatin-induced myopathy 2014 update was used to assign the likely OATP1B1 phenotype (normal function, intermediate function, and low function) and * allele nomenclature (Ramsey et al., 2014).

### Statistical Analysis

The frequencies of two SNPs of *SLCO1B1*, c.388A>G and c.521T>C, were checked for Hardy-Weinberg equilibrium using the R statistic version 3.6.1, the R Foundation for Statistical Computing. Fisher's Exact and Chi-square tests were used to determine the statistical difference between the minor alleles and haplotype frequencies between the geographical regions of Thailand using SPSS version 18.0 for Window, SPSS Inc., Chicago, IL, United States. A *p*-value of less than 0.05 was considered significant.

## Results

### Allele and Haplotype Frequencies of *SLCO1B1* in Thai Population

The allele frequencies of the non-synonymous polymorphic variants in the coding region c.388A>G (N130D) and c.521T>C (V174A) of *SLCO1B1* gene and haplotype frequencies of SLCO1B1*1a, *1b, *5, and *15 in 1,205 healthy Thai samples distributed over five regions of Thailand are shown in **Table 1**. All detected variations were in Hardy-Weinberg equilibrium (*p*>0.05). The allele frequencies of c.388A>G were similar among five regions. At the same time, the SNP c.521T>C showed the most frequency in Bangkok and the least in the Southern region, however, there were no significant differences in minor allele frequencies of these two SNPs among the five regions of Thailand. The most frequent haplotype was SLCO1B1*1b (frequency: 0.654), followed by *1a (frequency: 0.217), *15 (frequency: 0.128), and *5 (frequency: 0.001). We did not observe significant differences in haplotype frequencies among the five regions (**Table 1**).

**Table 1:** The minor allele frequencies of *SLCO1B1* c.388A>G (rs2306283) and c.521T>C (rs4149056) and the observed frequencies for selected *SLCO1B1* haplotypes in Thais distributed among the five regions of Thailand.

SLCO1B1	Total	Northern	Central	Northeastern	Southern	Bangkok	*p*-value
**Minor allele frequencies**							
**N**	**1,205**	**279**	**318**	**379**	**159**	**70**	
c.388A>G	0.782	0.805	0.769	0.792	0.755	0.757	0.328
c.521T>C	0.129	0.120	0.143	0.137	0.085	0.150	0.090
**Haplotype frequencies (%)**							
**N**	**2,410**	**558**	**636**	**758**	**318**	**140**	
*1a ^a^	21.74	19.53	22.96	20.84	24.21	24.29	0.382
*1b ^b^	65.39	68.46	62.74	65.44	67.30	60.71	0.187
*5 ^c^	0.08	0.00	0.16	0.00	0.31	0.00	0.450
*15 ^d^	12.78	12.01	14.15	13.72	8.18	15.00	0.071

**^a^**, *1a = wild type at all loci; **^b^**, *1b =rs2306283 G allele (A ancestral) (c.388A>G, p.N130D); **^c^**, *5 = rs4149056 Callele (T ancestral) (c.521T>C, p.V174A); ^d^, *15 = rs2306283 G allele (A ancestral) and rs4149056 C allele (T ancestral).

### The Phenotypes of OATP1B1 Transporter Based on *SLCO1B1* Diplotypes in Thai Population

The phenotypes of the OATP1B1 transporter have been assigned based on the diplotype at c.388A>G and c.521T>C of the *SLCO1B1* gene. The phenotype frequencies distributed over the five regions of Thailand and worldwide are shown in **Table 2**.

**Table 2:** OATP1B1 phenotypes based on *SLCO1B1* diplotypes in Thais distributed among the five regions of Thailand.

OATP1B1 phenotypes	*SLCO1B1* diplotypes	Phenotype frequencies (%)						
		Worldwide*^a^*	Total	Northern	Central	North eastern	Southern	Bangkok
Normal function	*1a/*1a *1a/*1b *1b/*1b	55 – 88	75.85	76.70	73.27	74.14	84.91	72.86
Intermediate function	*1a/*5 *1a/*15 *1b/*5 *1b/*15	11 – 36	22.57	22.58	24.84	24.27	13.21	24.29
Low function	*5/*5 *5/*15 *15/*15	0 – 6	1.58	0.72	1.89	1.58	1.89	2.86

**^a^**, Frequency of the polymorphism varies by ancestral group.

## Discussion

**Figure 1:**
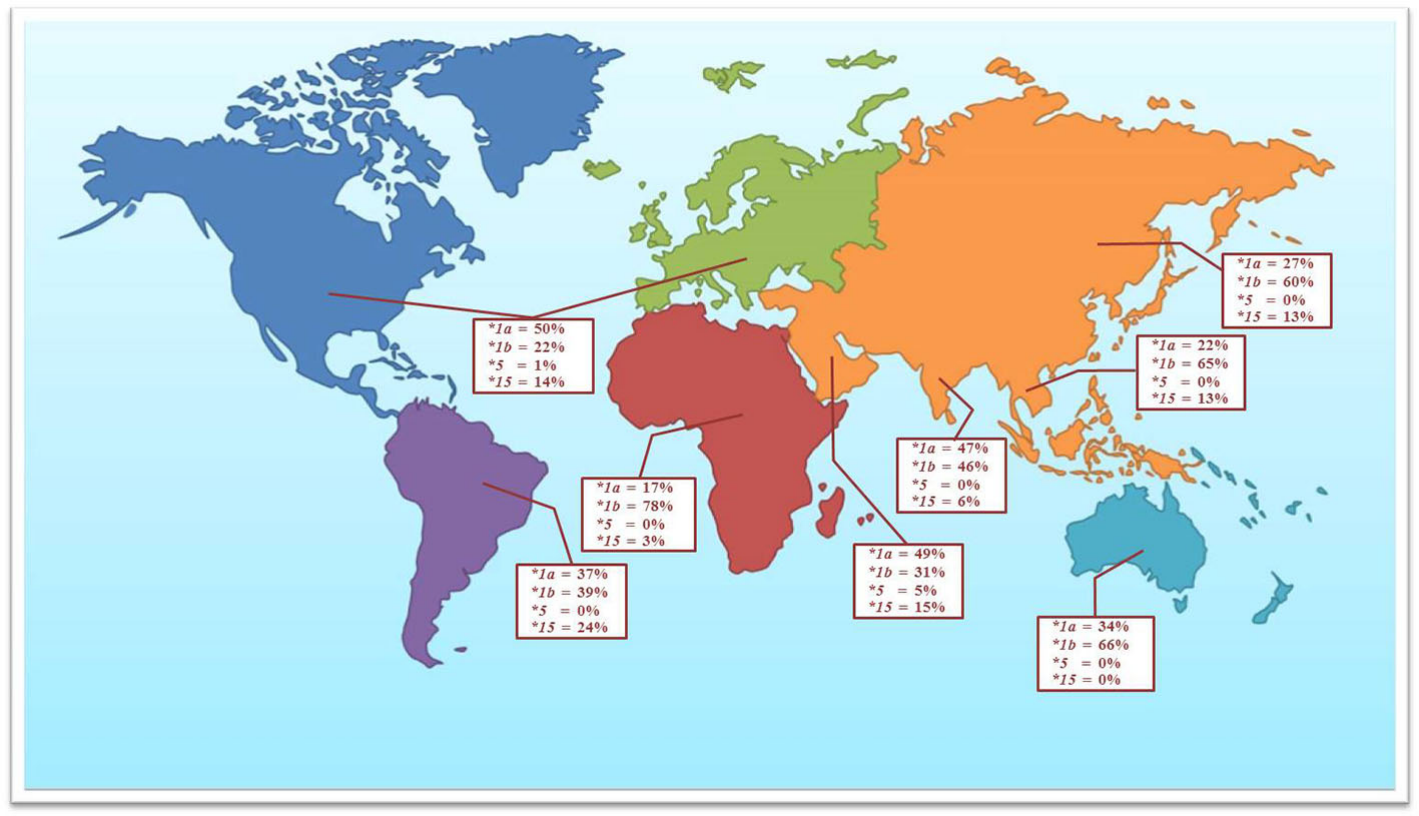
The observed frequencies for selected *SLCO1B1* haplotypes in the Thai population and other geographical groups (data adapted from Ramsey et al., 2014).

Numerous data have reported the genetic variations of the *SLCO1B1* gene for the determination of clinical drug response. The allele frequencies of the polymorphic variations and haplotype frequencies of the *SLCO1B1* gene, which are SLCO1B1*1a, SLCO1B1*1b, SLCO1B1*5, and SLCO1B1*15, have been studied in the various population groups. Among the published reports, *SLCO1B1* c.388A>G (N130D) and *SLCO1B1* c.521T>C (V174A) are the most commonly investigated SNPs in the various ethnic groups. This present study investigated the frequencies of these two common polymorphic variations in the Thai population distributed across five regions of Thailand. We found that allele frequencies of the c.388A>G and c.521T>C variants were close to the frequencies reported in other Asian populations, including Han Chinese, Japanese, Korean, and Vietnamese (Kim et al., 2008; Namgoong et al., 2015). When compared separately, the frequencies of c.388A>G were similar in Asian ancestry but showed differently in Asian Indians and Caucasians, while the frequencies of c.521T>C showed similarity in all ethnicity except Asian Indian and African ancestry (**Table 3** and **Figure 1**).

**Table 3:** The observed frequencies for selected *SLCO1B1* haplotypes in the Thai population and other geographical groups.

*SLCO1B1* haplotypes	Haplotype frequencies (%)							
	Thai	Asian*^a^*	SW Asian*^b^*	Middle Eastern	Oceania	Caucasian*^c^*	South/ Central American	African*^d^*
*1a	22	27	47	49	34	50	37	17
*1b	65	60	46	31	66	22	39	78
*5	0	0	0	5	0	1	0	0
*15	13	13	6	15	0	14	24	3

**^a^**, Asian = East Asian, Chinese, Japanese, Korean, Malays, and Vietnamese; **^b^**, SW Asian = South/Central Asian and Indian; **^c^**, Caucasian = American, European, Canadian, German, Finnish, French, Dutch, and Turkish; **^d^**, African = American, North African, Sub-Saharan Africa, Ugandan, and Tanzanian.

The most remarkable haplotypes of the *SLCO1B1* gene, SLCO1B1*5 and SLCO1B1*15, have been reported to reduce the number and function of the OATP1B1 transporter. These haplotypes not only alter the disposition of many therapeutic drugs, including various statins, but also affect the adverse events, especially statin-induced myotoxicity (0.3% in Thai population). Many reports have confirmed that the SLCO1B1*5 allele is associated with statin-induced myotoxicity in several populations (Hamann et al., 2013; Sakamoto and Kimura, 2013; Ramsey et al., 2014). Nevertheless, the frequency of SLCO1B1*5 haplotype in Asian population was very low when compared with other geographical groups (**Figure 1**). Published research on haplotype frequencies of the SLCO1B1*5 have shown the prevalence of 1.2% in a Chinese population, 0.7% in Japanese, and absent in Korean, and Vietnamese. We observed the SLCO1B1*5 haplotype having a frequency of 0.08% in the Thai population. SLCO1B1*1b is the most abundant haplotype in Asian populations (approximately 55% to 70%). The frequency of SLCO1B1*15 in our study showed a similar range compared to the Asian populations but lower when compared with South American populations (Nozawa et al., 2002; Kim et al., 2008).

We observed similar diplotype frequencies of the OATP1B1 transporter in the Thai population compared to previous reports in Asian populations, including Han Chinese, Japanese, Korean, and Vietnamese (Nozawa et al., 2002; Kim et al., 2008). The *SLCO1B1* phenotypes based on diplotypes in the Thai population were in agreement with the comprehensive data of the function of the OATP1B1 transporter (**Table 3**).

In conclusion, frequencies of the *SLCO1B1* variants and the subsequent OATP1B1 activity in a large cohort of Thais can provide important information for the guidance of personalized drug therapy.

## Data Availability Statement

The raw data supporting the conclusions of this article will be made available by the authors, without undue reservation, to any qualified researcher.

## Ethics Statement

The studies involving human participants were reviewed and approved by Committee on Human Rights Related to Reseach Involving Human Subjects, Faculty of Medicine Ramathibodi Hospital, Mahidol University. Written informed consent for participation was not required for this study in accordance with the national legislation and the institutional requirements.

## Author Contributions 

CN and CS were responsible for analysis, interpretation of data, and final approval of the manuscript. JW and CD were responsible for concept and design. PS was responsible for the analysis of data. SW and CS were responsible for supervising the overall conduct of the study.

## Conflict of Interest

The authors declare that this study was conducted in the absence of any commercial or financial relationships that can be a potential conflict of interest.

## References

[B1] Alam K. Crowe A. Wang X. Zhang P. Ding K. Li L. (2018). Regulation of Organic Anion Transporting Polypeptides (OATP) 1B1- and OATP1B3-Mediated Transport: An Updated Review in the Context of OATP-Mediated Drug-Drug Interactions. Int. J. Mol. Sci. 19 (3). 10.3390/ijms19030855 PMC5877716 29538325

[B2] Albayda J. Christopher-Stine L. (2014). Identifying statin-associated autoimmune necrotizing myopathy. Cleve. Clin. J. Med. 81 (12), 736–741. 10.3949/ccjm.81a.13158 25452351

[B3] Bhardwaj S. Selvarajah S. Schneider E. B. (2013). Muscular effects of statins in the elderly female: a review. Clin. Interv. Aging 8, 47–59. 10.2147/CIA.S29686 23355775 PMC3552608

[B4] Dandona S. (2014). Cardiovascular drugs and the genetic response. Methodist. Debakey. Cardiovasc. J. 10 (1), 13–17. 10.14797/mdcj-10-1-13 24932357 PMC4051328

[B5] Ghatak A. Faheem O. Thompson P. D. (2010). The genetics of statin-induced myopathy. Atherosclerosis 210 (2), 337–343. 10.1016/j.atherosclerosis.2009.11.033 20042189

[B6] Gong I. Y. Kim R. B. (2013). Impact of genetic variation in OATP transporters to drug disposition and response. Drug Metab. Pharmacokinet. 28 (1), 4–18. 10.2133/dmpk.DMPK-12-RV-099 23047721

[B7] Hamann P. D. Cooper R. G. McHugh N. J. Chinoy H. (2013). Statin-induced necrotizing myositis - a discrete autoimmune entity within the statin-induced myopathy spectrum. Autoimmun. Rev. 12 (12), 1177–1181. 10.1016/j.autrev.2013.07.001 23851103 PMC4589155

[B8] Ho W. F. Koo S. H. Yee J. Y. Lee E. J. (2008). Genetic variations of the SLCO1B1 gene in the Chinese, Malay and Indian populations of Singapore. Drug Metab. Pharmacokinet. 23 (6), 476–482. 10.2133/dmpk.23.476 19122343

[B9] Hu M. Mak V. W. Tomlinson B. (2012). Intronic variants in SLCO1B1 related to statin-induced myopathy are associated with the low-density lipoprotein cholesterol response to statins in Chinese patients with hyperlipidaemia. Pharmacogenet. Genomics 22 (11), 803–806. 10.1097/FPC.0b013e3283557c98 22668755

[B10] Kim S. R. Saito Y. Sai K. Kurose K. Maekawa K. Kaniwa N. (2007). Genetic variations and frequencies of major haplotypes in SLCO1B1 encoding the transporter OATP1B1 in Japanese subjects: SLCO1B1*17 is more prevalent than *15. Drug Metab. Pharmacokinet. 22 (6), 456–461. 10.2133/dmpk.22.456 18159134

[B11] Kim E. Y. Cho D. Y. Shin H. J. Lee S. S. Shon J. H. Shin J. G. (2008). Duplex pyrosequencing assay of the 388A>G and 521T>C SLCO1B1 polymorphisms in three Asian populations. Clin. Chim. Acta 388 (1-2), 68–72. 10.1016/j.cca.2007.10.010 17996736

[B12] Link E. Parish S. Armitage J. Bowman L. Heath S. Matsuda F. (2008). SLCO1B1 variants and statin-induced myopathy–a genomewide study. N Engl. J. Med. 359 (8), 789–799. 10.1056/NEJMoa0801936 18650507

[B13] Maeda K. (2015). Organic anion transporting polypeptide (OATP)1B1 and OATP1B3 as important regulators of the pharmacokinetics of substrate drugs. Biol. Pharm. Bull. 38 (2), 155–168. 10.1248/bpb.b14-00767 25747975

[B14] Mammen A. L. Amato A. A. (2010). Statin myopathy: a review of recent progress. Curr. Opin. Rheumatol. 22 (6), 644–650. 10.1097/BOR.0b013e32833f0fc7 20827205

[B15] Mastaglia F. L. Needham M. (2012). Update on toxic myopathies. Curr. Neurol. Neurosci. Rep. 12 (1), 54–61. 10.1007/s11910-011-0232-9 21968786

[B16] Mastaglia F. L. (2010). Iatrogenic myopathies. Curr. Opin. Neurol. 23 (5), 445–449. 10.1097/WCO.0b013e32833c2054 20581681

[B17] Muntean D. M. Thompson P. D. Catapano A. L. Stasiolek M. Fabis J. Muntner P. (2017). Statin-associated myopathy and the quest for biomarkers: can we effectively predict statin-associated muscle symptoms? Drug Discovery Today 22 (1), 85–96. 10.1016/j.drudis.2016.09.001 27634340

[B18] Namgoong S. Cheong H. S. Kim J. O. Kim L. H. Na H. S. Koh I. S. (2015). Comparison of genetic variations of the SLCO1B1, SLCO1B3, and SLCO2B1 genes among five ethnic groups. Environ. Toxicol. Pharmacol. 40 (3), 692–697. 10.1016/j.etap.2015.08.033 26409184

[B19] Nozawa T. Nakajima M. Tamai I. Noda K. Nezu J. Sai Y. (2002). Genetic polymorphisms of human organic anion transporters OATP-C (SLC21A6) and OATP-B (SLC21A9): allele frequencies in the Japanese population and functional analysis. J. Pharmacol. Exp. Ther. 302 (2), 804–813. 10.1124/jpet.302.2.804 12130747

[B20] Rallidis L. S. Fountoulaki K. Anastasiou-Nana M. (2012). Managing the underestimated risk of statin-associated myopathy. Int. J. Cardiol. 159 (3), 169–176. 10.1016/j.ijcard.2011.07.048 21813193

[B21] Ramsey L. B. Johnson S. G. Caudle K. E. Haidar C. E. Voora D. Wilke R. A. (2014). The clinical pharmacogenetics implementation consortium guideline for SLCO1B1 and simvastatin-induced myopathy: 2014 update. Clin. Pharmacol. Ther. 96 (4), 423–428. 10.1038/clpt.2014.125 24918167 PMC4169720

[B22] Sakamoto K. Kimura J. (2013). Mechanism of Statin-Induced Rhabdomyolysis. J. Pharmacol. Sci. 123 (4), 289–294. 10.1254/jphs.13R06CP 24257439

[B23] Sathasivam S. (2012). Statin induced myotoxicity. Eur. J. Intern. Med. 23 (4), 317–324. 10.1016/j.ejim.2012.01.004 22560377

[B24] Shitara Y. Maeda K. Ikejiri K. Yoshida K. Horie T. Sugiyama Y. (2013). Clinical significance of organic anion transporting polypeptides (OATPs) in drug disposition: their roles in hepatic clearance and intestinal absorption. Biophar. Drug Dispos. 34 (1), 45–78. 10.1002/bdd.1823 23115084

[B25] Sirtori C. R. Mombelli G. Triolo M. Laaksonen R. (2012). Clinical response to statins: mechanism(s) of variable activity and adverse effects. Ann. Med. 44 (5), 419–432. 10.3109/07853890.2011.582135 21623698

[B26] Wongkittichote P. Sukasem C. Kikuchi A. Aekplakorn W. Jensen L. T. Kure S. (2013). Screening of SLC25A13 mutation in the Thai population. World J. Gastroenterol. 19 (43), 7735–7742. 10.3748/wjg.v19.i43.7735 24282362 PMC3837273

